# Glucose Deprivation Triggers Protein Kinase C-dependent β-Catenin Proteasomal Degradation*

**DOI:** 10.1074/jbc.M114.606756

**Published:** 2015-02-17

**Authors:** Seung-Won Choi, Jun-Kyu Song, Ye-Seal Yim, Ho-Geun Yun, Kyung-Hee Chun

**Affiliations:** From the ‡ Department of Biochemistry and Molecular Biology, Yonsei University College of Medicine, 50 Yonsei-ro, Seodaemun-gu, Seoul 120-752, Korea and; the § Brain Korea 21 Plus Project for Medical Science, Yonsei University, 50 Yonsei-ro, Seodaemun-gu, Seoul 120-752, Korea

**Keywords:** Autophagy, beta-Catenin, Glucose, Glycogen Synthase Kinase 3 (GSK3), Protein Kinase C (PKC)

## Abstract

Autophagy is a conserved process that contributes to cell homeostasis. It is well known that induction mainly occurs in response to nutrient starvation, such as starvation of amino acids and insulin, and its mechanisms have been extensively characterized. However, the mechanisms behind cellular glucose deprivation-induced autophagy are as of now poorly understood. In the present study, we determined a mechanism by which glucose deprivation induced the PKC-dependent proteasomal degradation of β-catenin, leading to autophagy. Glucose deprivation was shown to cause a sub-G_1_ transition and enhancement of the LC3-II protein levels, whereas β-catenin protein underwent degradation in a proteasome-dependent manner. Moreover, the inhibition of GSK3β was unable to abolish the glucose deprivation-mediated β-catenin degradation or up-regulation of LC3-II protein levels, which suggested GSK3β-independent protein degradation. Intriguingly, the inhibition of PKCα using a pharmacological inhibitor and transfection of siRNA for PKCα was observed to effectively block glucose deprivation-induced β-catenin degradation as well as the increase in LC3-II levels and the accumulation of a sub-G_1_ population. Together, our results demonstrated a molecular mechanism by which glucose deprivation can induce the GSK3β-independent protein degradation of β-catenin, leading to autophagy.

## Teaser

**Background:** Cellular glucose deprivation induces cell cycle arrest and autophagy through proteasomal degradation of β-catenin.

**Results:** Glucose deprivation-induced β-catenin degradation and autophagy were not affected by GSK3β.

**Conclusion:** Only inhibition of PKCα caused retardation of β-catenin degradation and autophagy.

**Significance:** Cellular glucose deprivation activates PKCα to induce both autophagy and degradation of β-catenin.

## Introduction

Autophagy is an evolutionarily catabolic process by which unnecessary organelles and dysfunctional cellular components are degraded in lysosomes (1, 2). The products resulting from breakdown are input to cellular metabolism, where they are used to generate energy and to build new proteins and membranes under starvation conditions (3). Three different forms of autophagy occur in most cells: 1) microautophagy, which consists of the direct engulfment of cytoplasmic components by lysosomes; 2) chaperon-mediated autophagy, which selectively degrades cytosolic proteins that contain KFERQ-like sequences; and 3) macroautophagy, the most important form of autophagy, whereby cytoplasmic components are surrounded by a double membrane to form an autophagosome, which finally fuses with lysosomes to form autolysosomes, wherein the cargo is degraded.

In the past several decades, many researchers have been interested in the regulation of autophagy, and the related effects of amino acid depletion have been extensively studied. However, the role of glucose has gained less attention despite its implication in diseases, such as diabetes and different types of cancer. Recently, β-catenin was shown to interact with LC3, with direct targeting for autophagic degradation during nutrient stress (4). In the absence of Wnt stimulation, a destruction complex composed of casein kinase 1α, glycogen synthase kinase 3β (GSK3β),^3^ Axin, and adenomatous polyposis coli is formed. In this complex, casein kinase 1α and GSK3β phosphorylate a cytosolic conserved Ser/Thr-rich sequence near the N terminus of β-catenin to generate a recognition site for the E3 ubiquitin ligase, β-TrCP (β-transducin repeat-containing protein), and degradation by the 26 S proteasome (5). Upon Wnt activation, the destruction complex is disassembled, inhibiting β-catenin degradation. Accumulated cytoplasmic β-catenin translocates into the nucleus, where it displaces the Groucho family of transcriptional repressors from lymphoid enhancer-binding factor and TCF and acts as a transcriptional co-activator for target genes (6). In the noncanonical Wnt pathway, phospholipase C-mediated increases in intracellular Ca^2+^ levels and Ca^2+^ fluxes lead to the activation of Ca^2+^/calmodulin-dependent protein kinase, protein kinase C (PKC), and nuclear factor of activated T cells. This signaling has been shown to mediate dorsoventral patterning and tissue separation in embryos and to oppose canonical Wnt/β-catenin signaling (7–10). In addition, the β-catenin pathway can be negatively regulated by Wnt5a signaling in a GSK3β-independent manner (11, 12). However, the relationship between glucose deprivation-mediated autophagy and the stability of β-catenin protein is poorly understood. Here, we demonstrate that glucose deprivation can induce autophagy by promoting the degradation of β-catenin. We further show that the degradation of β-catenin upon glucose deprivation is GSK3β-independent and involves the PKC-dependent pathway.

## MATERIALS AND METHODS

### 

#### 

##### Cell Culture and Glucose Deprivation

HEK293 (human embryonic kidney, ATCC catalog no. CRL-1573) and HFF-1 (human foreskin fibroblast, ATCC catalog no. SCRC-1041) cells were maintained in Dulbecco's modified Eagle's medium (4,500 mg/liter glucose; Welgene, Daegu, Republic of Korea) supplemented with 10% (v/v) fetal bovine serum (Cellgro, Manassas, VA) and 1% penicillin/streptomycin at 37 °C in a humidified 5% CO_2_ incubator. To evaluate the effects of glucose deprivation, cells were rinsed with phosphate-buffered saline (PBS) and then cultured in glucose-free Dulbecco's modified Eagle's medium (Welgene, Deagu, Republic of Korea) containing 10% (v/v) fetal bovine serum and 1% penicillin/streptomycin for up to 4 days.

##### Reagents and Antibodies

Lithium chloride (LiCl) and d-(+)-glucose were obtained from Sigma-Aldrich, whereas Go6976 and MG132 were purchased from Merck Millipore (Darmstadt, Germany). MLN-4924 was purchased from Active Biochem (Hong Kong, China). An anti-FLAG monoclonal antibody was purchased from Sigma-Aldrich. Anti-hemagglutinin (HA) polyclonal antibody, phospho-Rb (Ser-807/811), Rb, CDK2, CDK4, cyclin A, cyclin B, cyclin D, cyclin E, p21, p27, phospho-GSK3β (Ser-9), GSK3β, phospho-AMP-activated protein kinase (Thr-172), AMP-activated protein kinase, OGT, OGA, phospho-Akt (Thr-308), Akt, β-actin, β-catenin, and TCF4 antibodies were obtained from Santa Cruz Biotechnology, Inc. Antibodies for cytochrome *c*, caspase-3, caspase-8, caspase-9, phospho-mTOR (Ser-2481), phospho-mTOR (Ser-2448), mTOR, phospho-P70S6K (Thr-389), P70S6K, LC3, phospho-ERK1/2 (Thr-202/Tyr-204), and ERK were purchased from Cell Signaling Technology (Danvers, MA). Mono- and polyubiquitinated antibodies were obtained from Enzo Life Sciences (Farmingdale, NY). The horseradish peroxidase-conjugated secondary antibodies were from Bethyl Laboratories (Montgomery, AL).

##### siRNA and Reverse Transcription-PCR (RT-PCR)

Double-stranded small interfering RNAs (siRNAs) for human GSK3β and PKCα as well as scrambled control siRNA were purchased from Genolution (Seoul, Korea) and transfected using RNAiMAX (Life Technologies) as described previously (13, 14). Total RNA from HEK293 cells was isolated using the TRIzol reagent (Life Technologies), according to the method of previous studies (15, 16). The purity and concentration of the RNA samples were measured with a NanoDrop (Thermo Scientific, Hanover Park, IL), with *A*_260/280_ ratios ranging from 1.8 to 2.0 considered acceptable. Reverse transcription (RT) was performed using the ReverTra Ace qPCR RT kit (Toyobo Co., Ltd.) as described previously (17, 18). The RT-PCR analysis was performed using Ex-taq (TaKaRa Korea Biomedical Inc.), and samples were run on a TaKaRa PCR thermal cycler (TaKaRa). PCR was performed with the following oligonucleotides, targeting human cDNA sequences: β-actin, 5′-AGCCTCGCCTTTGCCGA-3′ (sense) and 5′-CTGGTGCCTGGGGCG-3′ (antisense); β-catenin, 5′-CTTGCTCAGGACAAGGAAGC-3′ (sense) and 5′-CCTGGGCACCAATATCAAGT-3′ (antisense). The sequences for human PKCα 1 and 2 and GSK3β siRNA and scrambled control siRNA were as follows: PKCα siRNA 1, 5′-GGAUCCAAACGGGCUUUCAGAUU-3′ (sense) and 5′-UCUGAAAGCCCGUUUGGAUCCUU-3′ (antisense); PKCα siRNA 2, 5′- AAAGGCUGAGGUUGCUGAU-3′ (sense) and 5′-AUCAGCAACCUCAGCCUUU-3′ (antisense); GSK3β siRNA, 5′-CACUGGUCACGUUUGGAAAUU-3′ (sense) and 5′-UUUCCAAACGUGACCAGUGUU-3′ (antisense); and Scrambled control siRNA, 5′-CGUACGCGGAAUACUUCGAUU-3′ (sense) and 5′-UCGAAGUAUUCCGCGUACGUU-3′ (antisense).

##### Plasmids and Transfection

The β-catenin wild-type, mutant (S33F, S37A, and T41A), and GSK3β expression constructs were generous gifts from Prof. J. I. Yook and Prof. H. K. Kim (Yonsei University). In addition, reporter plasmids were obtained from J. I. Yook. Cells were seeded the day before transfection, and transfection was carried out with the indicated plasmids or siRNA using Lipfectamine® 2000 and RNAiMAX (Life Technologies), as described previously (19, 20). Cells were harvested 2 days after transfection for other experiments.

##### Treatment with Pharmacological Inhibitors

LiCl (10 mm) was used for the inhibition of GSK3β, Go6976 (3 μm) was used for PKC inhibition, and MLN-4924 (20 μm) was used to inhibit NEED8-activating enzyme. The inhibitors were added 24 h after either transfection or glucose deprivation. Following 24 h of incubation with the inhibitors, cells were lysed for the luciferase assay, Western analysis, or PI staining, as described previously (21, 22).

##### Luciferase Assay

β-Catenin/TCF transcriptional activity was measured via the TOPFlash luciferase activity assay. FOPFlash, which contains mutant lymphoid enhancer-binding factor/TCF binding sites, was used as a negative control for pathway specificity. Cells were transfected with reporter constructs (TOPFlash and FOPFlash), an internal control (β-galactosidase), and the indicated plasmids in 12-well plates. The total amount of transfected DNA was kept constant by adding empty vector DNA. After 48 h, the cells were harvested, and luciferase activity was measured using a luciferase assay system (Promega, Sunnyvale, CA), as described previously (13, 23). The results were presented as relative luciferase activity. The histogram data were presented as the average ± S.D. from three independent transfections.

##### Cell Fractionation

HEK293 and HFF-1 cells were lysed in lysis buffer (10 mm HEPES (pH 7.9), 1.5 mm MgCl_2_, 10 mm KCl, 1 mm DTT, 0.2 mm PMSF, 0.1% Nonidet P-40, and protease inhibitor mixture solution) for 15 min at 4 °C. After centrifugation at 850 × *g* for 10 min, supernatants were collected to obtain the cytoplasmic proteins. The nuclear pellets were then washed in lysis buffer lacking Nonidet P-40 and then repelleted. The nuclear pellets were resuspended in extraction buffer (20 mm HEPES (pH 7.9), 25% glycerol, 420 mm NaCl, 0.2 mm EDTA, 1.5 mm MgCl_2_, 1 mm DTT, 0.2 mm PMSF, and protease inhibitor mixture solution) with vortexing and then incubated for 30 min at 4 °C. After centrifugation at 10,000 × *g*, the supernatants were transferred to fresh tubes. The protein concentrations were determined using the Qubit protein assay kit (Life Technologies). Equal amounts of denatured nuclear and cytoplasmic protein were then resolved on a 10% SDS-polyacrylamide gel and immunoblotted following the procedure detailed below.

##### Immunoblotting

Cell lysates were prepared with radioimmune precipitation buffer (1% Nonidet P-40, 0.1% SDS, 0.5% deoxycholate, 150 mm NaCl, and 50 mm Tris, pH 7.5) containing protease and phosphatase inhibitor mixture solutions (GenDEPOT, Barker, NY). Thirty micrograms of total protein from each lysate was resolved on 5–12% SDS-polyacrylamide gels and electrotransferred to polyvinylidene fluoride (PVDF) membranes. After blocking in phosphate-buffered saline and 0.05% Tween 20 (PBST) containing 5% skim milk for 2 h, the membranes were incubated with the indicated primary antibodies overnight at 4 °C. Visualization of the immunoblots was performed using an enhanced chemiluminescence detection kit from Bio-Rad.

##### Co-immunoprecipitation

Cells were washed in cold PBS and lysed on ice with immunoprecipitation (IP) lysis buffer (20 mm Tris (pH 7.5), 140 mm NaCl, 1 mm EDTA, and 1% Nonidet P-40 (v/v), with protease inhibitor and phosphatase inhibitor mixture solutions) for co-IP analysis. The samples for detection of ubiquitin were additionally heated at 100 °C for 5 min. The cell extracts were then centrifuged at 13,000 × *g* for 10 min at 4 °C, and the supernatants were cleared with Sepharose-labeled protein A/G (Santa Cruz Biotechnology) beads for 1 h. The beads were discarded after a 1-min centrifugation at 2,000 × *g*, after which the supernatants were incubated with new Sepharose-labeled protein A/G beads and 2 μg of rabbit polyclonal anti-β-catenin antibody and rocked overnight at 4 °C. IP specificity was controlled for by treatment with rabbit IgG (Santa Cruz Biotechnology) for 1 h at 4 °C. The beads were then centrifuged for 30 s at 2,000 × *g* and washed four times with IP lysis buffer. For protein elution, 10 μl of 5× SDS-sample buffer (60 mm Tris, pH 6.8, 25% glycerol, 2% SDS, 14.4 mm β-mercaptoethanol, and 0.1% bromphenol blue) was added, followed by boiling at 100 °C for 5 min. The supernatants were then obtained after brief centrifugation, and the protein-protein interactions were determined via Western blot analysis.

##### Cell Cycle Detection

Cells were trypsinized with 0.5 ml of 0.25% trypsin for 2 min, collected, and centrifuged at 400 × *g* for 5 min at 4 °C. The supernatants were then removed by aspiration, and the pellets were washed twice with precooled PBS and centrifuged at 400 × *g* for 5 min at 4 °C. Cells were resuspended with 1 ml of precooled 70% ethanol and fixed overnight at 4 °C. After removing the ethanol and adding 0.5 ml of staining solution (50 μg/ml PI, 100 μg/ml RNase A, and 0.2% Triton X-100), the cells were incubated at room temperature for 30 min in the dark. Cell cycle distribution was analyzed by flow cytometry (BD Biosciences), as described previously (24).

## RESULTS

### 

#### 

##### Autophagy, Cell Cycle Regulators, and Metabolism Markers Can Be Regulated by Glucose Deprivation

**FIGURE 1.**
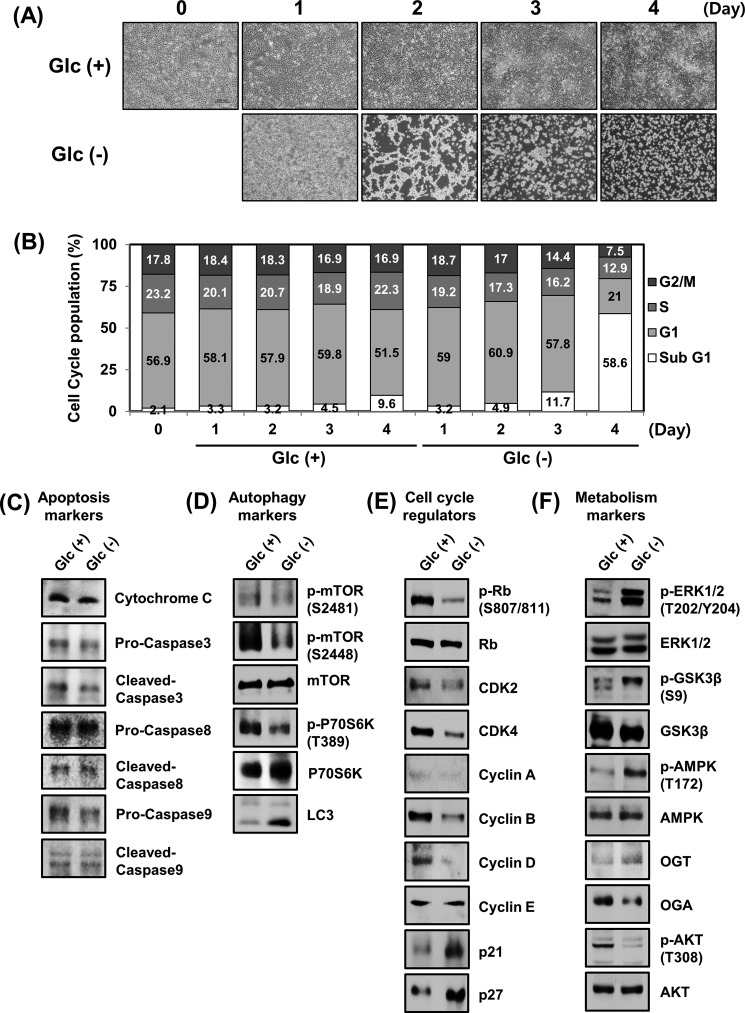
**Glucose deprivation induces autophagy in HEK293 cells.** *A*, cells were cultured in different conditions: cells cultured in DMEM with normal glucose (Glc(+)) and cells cultured in glucose-depleted DMEM (Glc(−)) for 0–4 days. Investigation of cell morphological changes under glucose deprivation conditions was performed using microscopy equipment. *Scale bars*, 200 μm. *B*, HEK293 cells were incubated in Glc(+) or Glc(−) conditions for 0–4 days. The cells were then collected and stained with PI and then analyzed by flow cytometry. HEK293 cells were incubated in Glc(+) or Glc(−) conditions. After 24 h of glucose deprivation, cells were harvested, and the indicated concentrations of endogenous protein were examined by immunoblotting. *C*, apoptosis markers; *D*, cell cycle regulators; *E*, metabolism markers; and *F*, autophagy markers.

To clarify the cellular phenomena of glucose deprivation, HEK293 and HFF-1 cells were incubated in glucose-depleted DMEM (Glc(−)) and then compared with those incubated in normal DMEM (Glc(+)). This revealed a time-dependent reduction in the cell number of both HEK293 and HFF-1 (data not shown), along with abnormal cellular phenotype under conditions of glucose deprivation (Fig. 1*A*). The effects of glucose deprivation on the cell cycle were also evaluated, by which glucose deprivation was found to lead to significant accumulation of sub-G_1_-positive cells in HEK293 (Fig. 1*B*). Conversely, HFF-1 showed more drastic movement to sub-G_1_ cell populations (data not shown). Next, we examined whether glucose deprivation modulated endogenous apoptosis, autophagy, cell cycle regulators, or the levels of metabolism-related proteins. No significant changes in apoptosis-related proteins were observed in HEK293 cells after glucose deprivation (Fig. 1*C*). In contrast, glucose deprivation led to cell cycle arrest through the down-regulation of phospho-Rb, CDK2, CDK4, and cyclin B as well as the up-regulation of p21 and p27 (Fig. 1*D*). Moreover, metabolic markers were also modulated by glucose deprivation. Specifically, the levels of phospho-ERK1/2, phospho-GSK3β, and phospho-AMP-activated protein kinase were significantly elevated after glucose deprivation, whereas phospho-Akt displayed a sharp drop due (Fig. 1*E*). Interestingly, we also found a decrease in the levels of phospho-mTOR and phospho-p70S6K after glucose deprivation, whereas LC3, a protein essential for autophagy, displayed a marked increase (Fig. 1*F*). Taken together, these results indicated that glucose deprivation induces cell cycle arrest and autophagy in HEK293 cells.

##### Glucose Deprivation Induces β-Catenin Protein Destabilization

**FIGURE 2.**
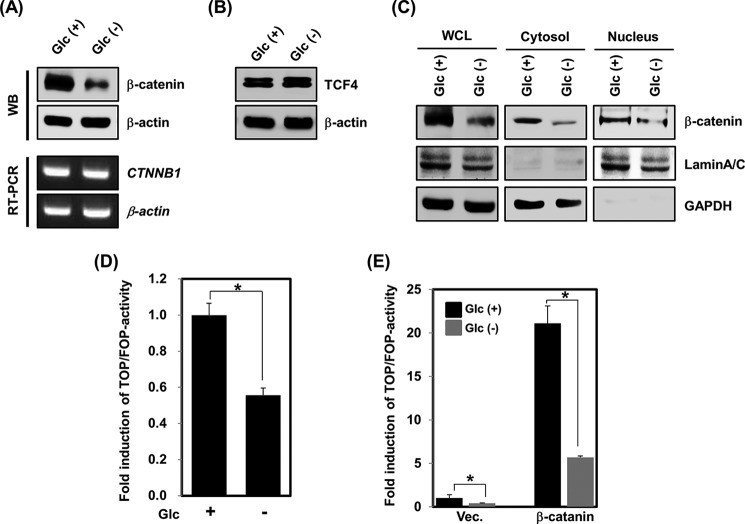
**β-Catenin protein stability is negatively regulated by glucose deprivation.** *A*, HEK293 cells were incubated in Glc(+) or Glc(−) conditions. Cells were harvested after 24 h of glucose deprivation, and the mRNA expression or protein levels of β-catenin and β-actin were examined either by RT-PCR or immunoblotting. *B*, HEK293 cells were incubated in Glc(+) or Glc(−) conditions for 24 h. The cell lysates were then analyzed by immunoblotting using an anti-TCF4 antibody. Equal loading was ensured using an anti-β-actin antibody. *C*, a cell fractionation assay of HEK293 cells was performed under Glc(+) or Glc(−) conditions for 24 h. Nuclear and cytoplasmic extracts were subjected to immunoblotting using anti-β-catenin, anti-lamin A/C, and anti-GAPDH antibodies. *D*, TOPFlash or FOPFlash reporter plasmids were transfected into HEK293 cells. At 24 h post-transfection, cells were incubated in Glc(+) or Glc(−) conditions for an additional 24 h and then processed for luciferase assays (means ± S.E. (*error bars*) from three independent experiments. *, *p* < 0.05. Both this assay and all subsequent luciferase assays in this paper were performed in triplicate and normalized to a co-transfected reporter construct encoding the β-galactosidase gene. *E*, an empty vector or HA-β-catenin construct was transfected into HEK293 cells in the presence of the TOPFlash or FOPFlash reporter gene for 24 h. After 24 h, cells were cultured in Glc(+) or Glc(−) conditions. Luciferase activity was measured from total cell extracts (means ± S.E. from three independent experiments; *, *p* < 0.05).

A recent study showed that the increase of apoptotic and autophagic cell death in response to treatment with β-catenin siRNA was due to reduced levels of β-catenin mRNA (25). Furthermore, in nutrient-rich conditions, β-catenin suppresses autophagy through the repression of p62, an autophagy adaptor protein (4). As an initial step, we sought to investigate whether glucose deprivation regulates the levels of β-catenin mRNA and/or protein. As a result, β-catenin protein was observed to be largely down-regulated by glucose deprivation compared with the control, whereas the mRNA expression was not altered in HEK293 cells (Fig. 2*A*). In contrast with β-catenin, the levels of TCF4 protein, a nuclear β-catenin binding partner, were not influenced by glucose deprivation (Fig. 2*B*). Thus, we next examined the nuclear and cytosolic distribution of β-catenin under conditions of glucose deprivation. As shown in Fig. 2*C*, the levels of β-catenin protein were substantially reduced in both compartments. To gain an understanding of how glucose deprivation inhibits β-catenin signaling, TOPFlash, which has eight copies of the consensus lymphoid enhancer-binding factor/TCF binding site, or the mutated FOPFlash reporters (26) were transfected into HEK293 cells. Glucose deprivation reduced the TOPFlash/FOPFlash ratio by 0.5-fold (Fig. 2*D*). In addition, overexpression of the β-catenin plasmid increased the TOPFlash/FOPFlash ratio by about 20-fold, whereas the reporter activity was markedly suppressed by glucose deprivation (Fig. 2*E*). These data suggest that glucose deprivation represses the β-catenin/TCF4 signaling pathway by promoting the degradation of β-catenin.

##### Glucose Deprivation Can Cause the Proteasome-dependent Degradation of β-Catenin

**FIGURE 3.**
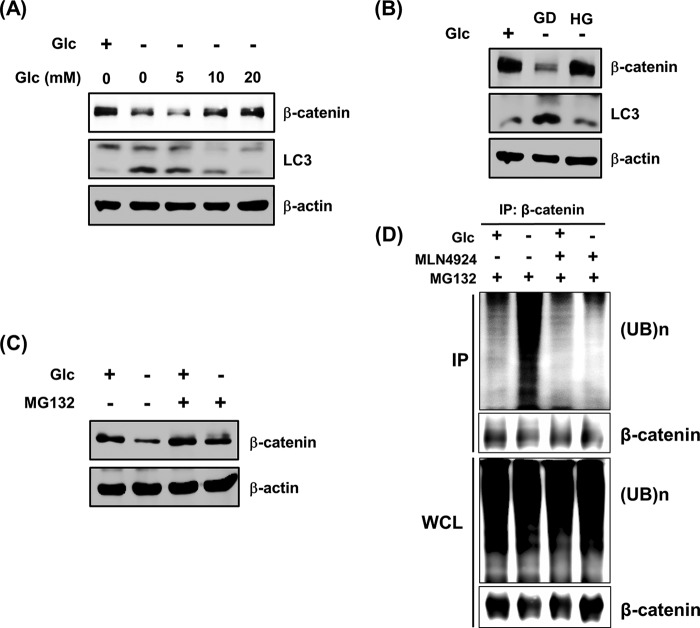
**Glucose deprivation-induced β-catenin degradation is triggered in a proteasome-dependent manner.** *A*, glucose-deprived HEK293 cells were incubated with the indicated dose of glucose. After 24 h, cells were harvested, and immunoblotting was performed using anti-β-catenin and anti-LC3 antibodies. Equal loading was ensured by using an anti-β-actin antibody. *B*, HEK293 cells were incubated in Glc(+) or Glc(−) conditions for 12 h. The glucose-deprived HEK293 cells were then cultured with (*HG*) or without (*GD*) glucose DMEM. After 12 h, cell lysates were analyzed by immunoblotting using anti-β-catenin and anti-LC3 antibodies. Equal loading was ensured using an anti-β-actin antibody. *C*, HEK293 cells were incubated in Glc(+) or Glc(−) conditions for 10 h. After 2 h of glucose deprivation, the cells were treated with 10 μm MG132 for 8 h and lysed. Total cell lysates were analyzed by immunoblotting using an anti-β-catenin antibody. Equal loading was evaluated using an anti-β-actin antibody. *D*, HEK293 cells were incubated in Glc(+) or Glc(−) conditions for 24 h. After 16 h of glucose deprivation, cells were exposed to 10 μm MG132 and 20 μm MLN4924 for the next 8 h of incubation. Cell lysates were boiled at 95 °C for 5 min and then subjected to immunoprecipitation with antibodies raised against β-catenin, followed by immunoblotting with ubiquitin (*UB*). Input cell lysates were analyzed for ubiquitin and β-catenin by immunoblotting.

As shown in Fig. 1*D*, HEK293 cells exhibited an increase in LC3 protein levels in response to glucose deprivation. Thus, we examined whether the administration of glucose can block the regulation of β-catenin and LC3 protein levels due to glucose deprivation. Glucose deprivation caused substantial decrease of β-catenin and increase of LC3-II protein. Conversely, these changes were dose-dependently reversed by treatment with d-glucose treatment (Fig. 3*A*). Consistent with these results, when glucose-depleted culture medium was replaced with medium containing 25 mm glucose, the levels of β-catenin and LC3-II protein were completely recovered to control levels (Fig. 3*B*). To examine whether the glucose deprivation-induced degradation of β-catenin is mediated by the proteasome, MG132, a potent and cell-permeable proteasome inhibitor, was employed. Marked attenuation of β-catenin degradation was observed in cells treated with MG132 (Fig. 3*C*). Furthermore, glucose deprivation was also found to cause substantially elevated levels of β-catenin ubiquitination. Moreover, the glucose deprivation-induced ubiquitination of β-catenin was completely blocked by treatment with the SCF complex inhibitor, MLN4924 (Fig. 3*D*). The SCF complex is a multiprotein E3 ubiquitin ligase complex that has important roles in the ubiquitination of proteins involved in the cell cycle and also marks various other cellular proteins for destruction (27). These results indicate that glucose deprivation can promote the proteasome-dependent degradation of β-catenin, triggering β-catenin ubiquitination.

##### Glucose Deprivation Promotes β-Catenin Degradation in a GSK3β-independent Manner

**FIGURE 4.**
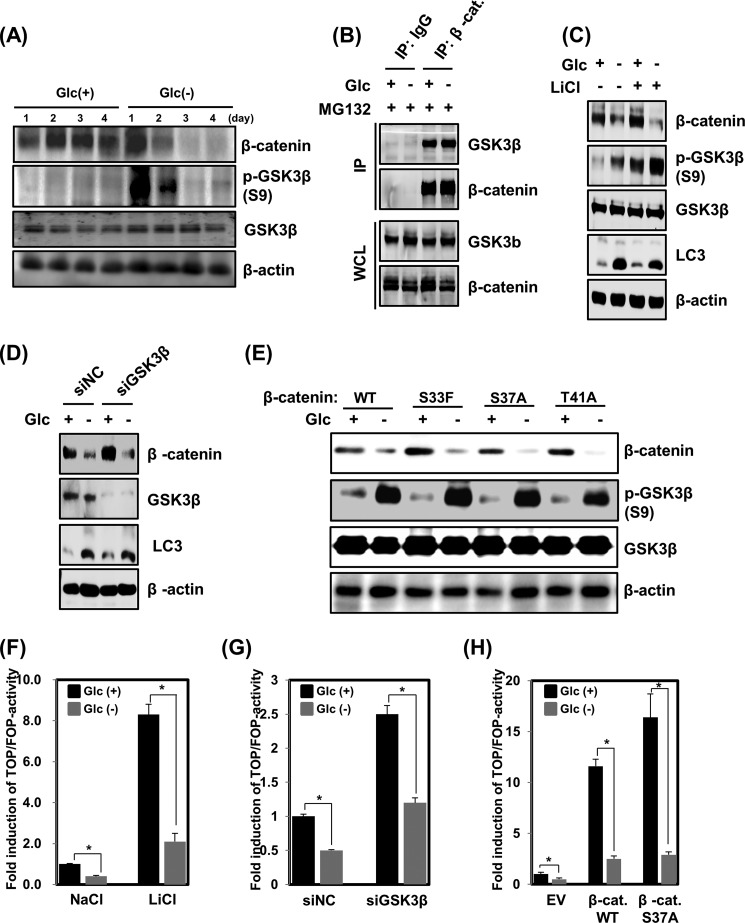
**GSK3β is not required for glucose deprivation-induced β-catenin degradation.** *A*, HEK293 cells were incubated in Glc(+) or Glc(−) DMEM for 24 h. Total cell lysates were analyzed for β-catenin, phospho-GSK3β, and GSK3β by immunoblotting. Equal loading was ensured using an anti-β-actin antibody. *B*, to assess the interaction between β-catenin and GSK3β under Glc(−) conditions, immunoprecipitation experiments were performed on HEK293 cell extracts. HEK293 cells were incubated in Glc(+) or Glc(−) DMEM for 24 h. After 16 h of glucose deprivation, the cells were treated with 10 μm MG132 for the final 8 h, after which cell lysates were subjected to immunoprecipitation with a β-catenin antibody, followed by GSK3β immunoblotting. Input lysates were analyzed for β-catenin and GSK3β by immunoblotting. *C*, HEK293 cells were cultured in Glc(+) or Glc(−) DMEM for 24 h in the presence or absence of 10 mm LiCl. The levels of endogenous β-catenin, phospho-GSK3β, GSK3β, and LC3 were evaluated by immunoblotting. Equal amounts of total protein were loaded and analyzed according to β-actin levels by immunoblotting. *D*, small interfering RNAs for scrambled control (*siNC*) or GSK3β (*siGSK3*β) were transfected into HEK293 cells for 48 h. After 24 h of transfection, the cells were incubated in Glc(+) or Glc(−) DMEM for an additional 24 h. Cell lysates were then analyzed by immunoblotting using anti-β-catenin, anti-GSK3β, and anti-LC3 antibodies. Equal loading of samples was ensured using an anti-β-actin antibody. *E*, HEK293 cells were transfected with WT or one of the S33F, S37F, or T41A mutant β-catenin expression constructs for 48 h. At 24 h post-transfection, cells were incubated in Glc(+) or Glc(−) DMEM for an additional 24 h. Cell lysates were analyzed by immunoblotting using anti-β-catenin, anti-phospho-GSK3β, and anti-GSK3β antibodies. Equal loading was ensured using an anti-β-actin antibody. *F*, TOFFlash or FOPFlash reporter genes were transfected into HEK293 cells for 48 h. At 24 h post-transfection, cells were incubated in Glc(+) or Glc(−) DMEM in the absence or presence of 10 mm LiCl. After an additional 24 h, luciferase assays were performed (means ± S.E. (*error bars*) from three independent experiments; *, *p* < 0.05). *G*, HEK293 cells were co-transfected with TOPFlash or FOPFlash reporter genes plus either scrambled siRNA or GSK3β siRNA for 48 h. At 24 h post-transfection, cells were cultured with normal DMEM or glucose-depleted DMEM for 24 h and then harvested. Cell extracts were subsequently processed for luciferase activity (means ± S.E. from three independent experiments; *, *p* < 0.05). *H*, HEK293 cells were co-transfected with TOPFlash or FOPFlash reporter genes plus either an empty vector, HA-β-catenin WT, or HA-β-catenin S37A. Transfected cells were cultured in Glc(+) or Glc(−) DMEM for the last 24 h of incubation and subsequently assayed for luciferase activity (means ± S.E. from three independent experiments; *, *p* < 0.05).

Because GSK3β is known to be mediated by β-catenin degradation (28), we next investigated the possibility that GSK3β could be involved in regulation of the observed reduction in β-catenin protein levels. The levels of phospho-GSK3β (Ser-9) were found to be significantly elevated until 48 h after glucose deprivation, whereas the levels of β-catenin protein were still reduced in both HEK293 and HFF-1 cells (Fig. 4*A*). However, the protein interactions between β-catenin and GSK3β were not inhibited by glucose deprivation (Fig. 4*B*). These results suggest that the inactivation of GSK3β, which is elevated, interacts with β-catenin after glucose deprivation, but such interaction did not influence β-catenin degradation. Next, it was confirmed that GSK3β activity was not associated with the protein stability of β-catenin under glucose deprivation. To accomplish this, glucose-depleted HEK293 cells were treated with LiCl, a direct inhibitor of GSK3β. LiCl increased the levels of phospho-GSK3β in the presence or absence of glucose. Nevertheless, significant destabilization of β-catenin was observed after glucose deprivation (Fig. 4*C*). Moreover, an RNA knockdown of GSK3β using siRNA increased the levels of endogenous β-catenin in normal glucose conditions but reduced the stability in the glucose-deprived state (Fig. 4*D*). Interestingly, as shown in Fig. 4, *C* and *D*, the levels of LC3-II protein, which were enhanced after glucose deprivation, were not reduced by the inhibition of GSK3β via either LiCl treatment or RNA knockdown. These results suggest that GSK3β was not associated with glucose deprivation-induced autophagy and β-catenin degradation. Finally, phosphorylation resistance mutants of β-catenin (S33F, S37A, and T41A) that had abolished GSK3β-dependent phosphorylation of β-catenin at Ser-33 or -37 and Thr-41, which is required for β-catenin degradation, were also employed in analysis (29, 30). Accordingly, these mutants were not destabilized by GSK3β. However, when wild-type β-catenin or the S33F, S37A, and T41A mutants were expressed in HEK293 cells, all were found to be destabilized under glucose-free conditions (Fig. 4*E*). Because glucose deprivation induced GSK3β levels independent of activity on β-catenin, we next probed the possibility that glucose deprivation might suppress β-catenin-responsive reporter activity in a GSK3β-independent manner. To this end, HEK293 cells were incubated with LiCl in the presence or absence of glucose deprivation. As shown in Fig. 4*F*, LiCl treatment caused a marked increase in TOPFlash/FOPFlash reporter activity, whereas the activity was effectively suppressed by glucose deprivation. Furthermore, the glucose deprivation-mediated suppression of TOPFlash/FOPFlash reporter activity could not be attenuated by an RNA knockdown of GSK3β (Fig. 4*G*) or by overexpression of the β-catenin S37A mutant (Fig. 4*H*).

These results indicated that β-catenin degradation induced upon glucose deprivation may be insufficiently activated by a β-catenin-responsive reporter gene. Taken together, these results indicate that GSK3β is not necessary for the glucose deprivation-triggered degradation of β-catenin.

##### Inhibition of GSK3β Does Not Antagonize Glucose Deprivation-induced Abnormal Cell Morphology and Increase of Sub-G_1_ Population

**FIGURE 5.**
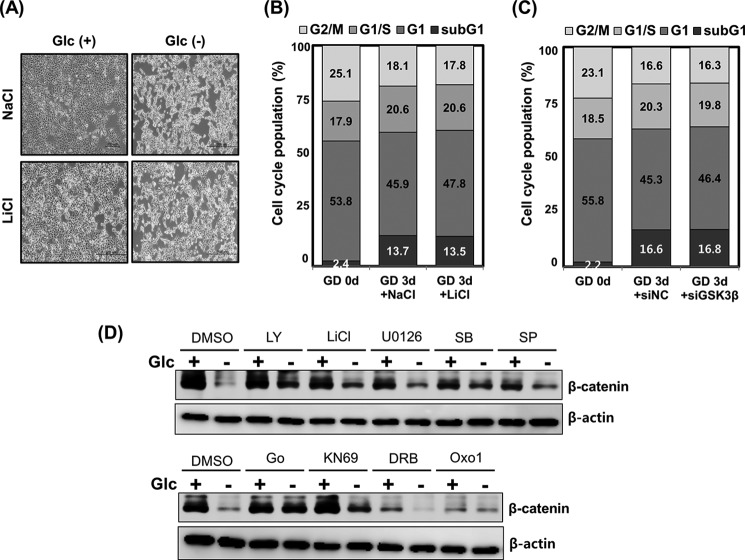
**GSK3β is not involved in the glucose deprivation-mediated increase of the sub-G_1_ population.** *A*, HEK293 cells were cultured in Glc(+) or Glc(−) DMEM for 24 h in the presence or absence of 10 mm LiCl. The investigation of cell morphological changes under glucose-deprived conditions was performed using microscopy equipment. *Scale bars*, 200 μm. *B*, HEK293 cells were incubated in Glc(+) or Glc(−) DMEM for 24 h in the presence or absence of 10 mm LiCl. After treatment, the cells were collected and stained with PI and then analyzed by flow cytometry. *C*, siRNAs for control or GSK3β were transfected into HEK293 cells for 48 h. At 24 h post-transfection, cells were incubated in Glc(+) or Glc(−) DMEM for an additional 72 h. Cell lysates were collected sequentially, stained with PI, and then analyzed by flow cytometry. *D*, HEK293 cells were incubated in Glc(+) or Glc(−) DMEM for 24 h in the presence or absence of kinase inhibitors: 5 μm LY294002 (*LY*)-PI3K inhibitor, 10 mm LiCl-GSK3β inhibitor, 10 μm U0126-MEK inhibitor, 20 μm SB203580 (*SB*)-p38 inhibitor, 10 μm SP600125 (*SP*)-JNK inhibitor, 3 μm Go6976 (*Go*)-PKCα inhibitor, 10 μm KN69-Ca^2+^/calmodulin-dependent protein kinase inhibitor, 100 μm 5,6-dichloro-1-β-d-ribofuranosyl-benzimidazole (*DRB*)-RNA polymerase inhibitor II, and 5 μm 2-(3-benzylamino-2-oxo-1,2-dihydropyridin-1-yl)-*N*-(3,4-dichlorobenzyl)acetamide (*Oxo1*)-glycogen phosphorylase inhibitor. Levels of β-catenin protein were evaluated with immunoblotting. Equal amounts of total protein were loaded and analyzed according to β-actin levels.

Because the results obtained so far suggested that GSK3β is not critical for glucose deprivation-mediated β-catenin degradation and increase in LC3 protein levels, we next asked whether it plays an essential role in autophagy-induced cell death. Toward this end, glucose-depleted HEK293 cells were treated with LiCl. As shown in Fig. 5*A*, the glucose deprivation-induced abnormal cell morphology could not be rescued through the inhibition of GSK3β via LiCl treatment.

PI staining was next performed to address whether GSK3β modulates glucose deprivation-mediated cell death. Consistent with the results from the cell morphology assay, the PI staining assay revealed that inhibition of GSK3β by treatment with LiCl did not affect the up-regulation of the sub-G_1_ population upon glucose deprivation (Fig. 5*B*). In addition, the enhancement of the sub-G_1_ population also showed no attenuation upon knockdown of GSK3β using siRNA (Fig. 5*C*). Taken together, these results clearly show that GSK3β is not required for glucose deprivation-mediated autophagy and β-catenin degradation.

##### PKCα Acts as a Regulator of β-Catenin Degradation and Autophagy Triggered by Glucose Deprivation

**FIGURE 6.**
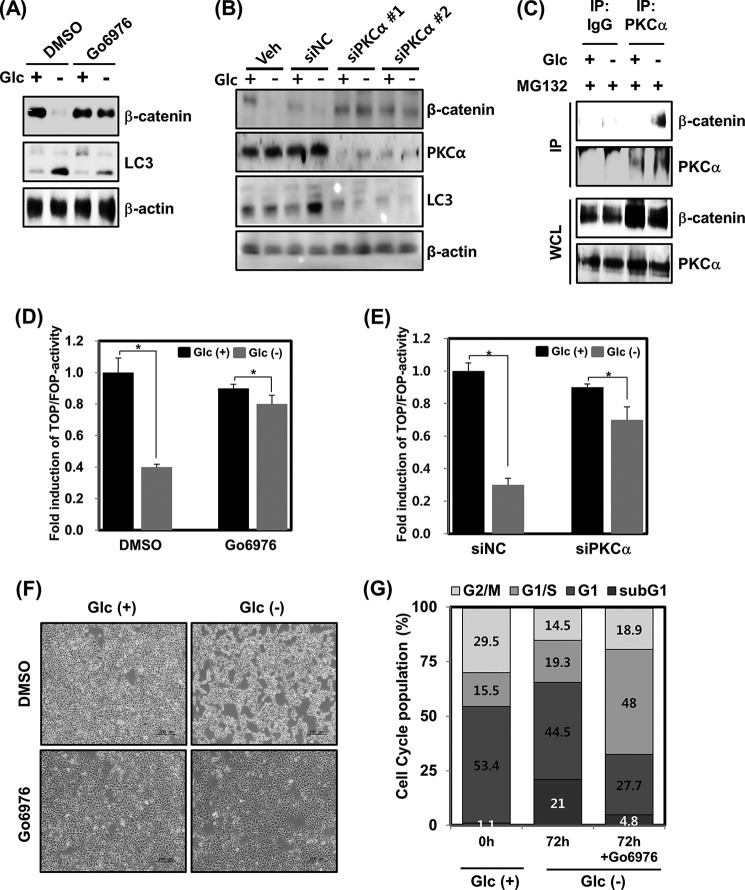
**PKCα mediates glucose deprivation-induced autophagy.** *A*, HEK293 cells were incubated in Glc(+) or Glc(−) DMEM for 24 h in the presence or absence of 3 μm Go6976. The levels of β-catenin and LC3 protein were examined by immunoblotting. Equal amounts of total protein were loaded and analyzed according to β-actin levels. *B*, HEK293 cells were transfected with a scrambled siRNA (*siNC*) or with two different sequences of PKCα siRNA (siPKCα 1 or 2). At 28 h post-transfection, the cells were harvested, and the lysates were analyzed by immunoblotting with anti-β-catenin, anti-LC, anti-PKCα, or β-actin (loading control) antibodies. *C*, HEK293 cells were incubated in Glc(+) or Glc(−) DMEM. At 16 h after glucose deprivation, the cells were treated with 10 μm MG132 for 8 h and harvested, and PKCα was immunoprecipitated with an anti-PKCα antibody. Immunoprecipitates were analyzed by immunoblotting with anti-β-catenin and anti-PKCα antibodies. *D*, TOFFlash or FOPFlash reporter genes were transfected into HEK293 cells for 48 h. At 24 h post-transfection, cells were incubated in Glc(+) or Glc(−) DMEM in the presence or absence of 3 μm Go6976. After an additional 24 h, luciferase assays were performed (means ± S.E. (*error bars*) from three independent experiments; *, *p* < 0.05). *E*, HEK293 cells were co-transfected with TOPFlash or FOPFlash reporter genes, plus either scrambled siRNA or PKCα 1 siRNA for 48 h. At 24 h post-transfection, cells were cultured with normal DMEM or glucose-depleted DMEM for an additional 24 h and then harvested. Cell extracts were subsequently processed for luciferase activity (means ± S.E. from three independent experiments; *, *p* < 0.05). *F*, HEK293 cells were cultured in Glc(+) or Glc(−) DMEM for 24 h in the presence or absence of 3 μm Go6976. The investigation of cell morphological changes under glucose deprivation conditions was performed using microscopy equipment. *Scale bars*, 200 μm. *G*, HEK293 cells were incubated in Glc(+) or Glc(−) DMEM for 72 h in the presence or absence of 3 μm Go6976. After treatment, the cells were collected and stained with PI and then analyzed by flow cytometry.

To determine which kinase affects the degradation of β-catenin upon glucose deprivation, cells were treated with several kinds of kinase inhibitors, including PKC, Ca^2+^/calmodulin-dependent protein kinase II, mitogen-activated protein kinase kinase 1/2 (MEK1/2), p38, and c-Jun N-terminal kinase (JNK), which have been indicated in various β-catenin regulation pathways (31–39). Through this analysis, it was found that Ca^2+^/calmodulin-dependent protein kinase, MEK1/2, p38, and JNK may not be closely associated with glucose deprivation-induced β-catenin destabilization (Fig. 5*D*). However, the observed β-catenin degradation was remarkably blocked by pharmacological inhibition of PKC using Go6976 (Figs. 5*D* and 6*A*). Consistent with this finding, a PKCα knockdown using siRNA resulted in the successful blockage of β-catenin degradation upon glucose deprivation (Fig. 6*B*). Intriguingly, the elevation of LC3-II protein levels observed upon glucose deprivation was also substantially inhibited by PKC inhibition using Go6976 (Fig. 6*A*) or by transfection with PKCα siRNA 1 or 2 (Fig. 6*B*). The possibility that glucose deprivation may regulate the interaction between β-catenin and PKCα was thus explored. The results revealed that PKCα could not form a complex with β-catenin under normal glucose conditions, but remarkably, formation of a protein complex between β-catenin and PKCα was observed after glucose deprivation (Fig. 6*C*). These results indicate that PKCα is engaged in the glucose deprivation-induced proteasomal degradation of β-catenin.

To further elucidate the relationship between PKC and the glucose deprivation-induced β-catenin degradation, we next investigated whether PKC inhibited the suppression of the TOPFlash/FOPFlash activity triggered by glucose deprivation. The reduction of TOPFlash/FOPFlash reporter activity observed after glucose deprivation was found to be effectively abolished by pharmacological inhibition of PKC via Go6976 (Fig. 6*D*) or by RNA knockdown of PKCα (Fig. 6*E*). In addition, Go6976 treatment also displayed efficient rescue of the abnormal cell morphology induced by glucose deprivation (Fig. 6*F*), whereas the sub-G_1_ population increase upon glucose deprivation was also sufficiently down-regulated by Go6976 treatment (Fig. 6*G*). Taken together, PKCα-mediated β-catenin degradation and the stabilization of LC3-II triggered by glucose deprivation may be an essential process for autophagy.

## DISCUSSION

Autophagy is a major contributor to cellular metabolism. It provides internal nutrients and is an essential means of refreshing and remodeling cells (40). The induction of autophagy by nutrient starvation, including lack of insulin and amino acids, has been extensively investigated, whereas insufficient attention has been given to glucose deprivation. In the present study, we observed that glucose deprivation significantly increased the levels of LC3-II protein and inhibited mTOR phosphorylation, which ultimately led to autophagy (Fig. 1*F*). Glucose deprivation also increased the expression of cell cycle inhibitors, such as p16^Ink4a^, p21^Cip1^, and p27^Kip1^, which represents a state of cell cycle arrest and cellular senescence. These results indicate that cells may undergo cell cycle arrest in response to early glucose deprivation, whereas senescent cells later transition to autophagy under conditions of glucose deprivation. Interestingly, glioblastoma cells have been reported to undergo apoptotic cell death in conditions of glucose deprivation, which was caused by oxidative stress (41). In tumor cells, inhibition of glucose metabolism causes death receptor-triggered apoptosis via the enhancement of death-inducing signaling complex formation and procaspase processing (42). These results indicate that glucose deprivation also induces apoptosis in cancer cells. However, not all cell death induced by glucose deprivation results in apoptosis, because the apoptotic markers, including cytochrome *c* and cleaved caspase-3, -8, and -9, were not altered by glucose deprivation in our system. One important finding herein is that glucose deprivation promoted the degradation of β-catenin through a GSK3β-independent pathway. With canonical Wnt activation, β-catenin is known to be stabilized by the inhibition of phosphorylation by GSK3β (43). Our result revealed that phosphorylation of GSK3β at Ser-9 was significantly increased after glucose deprivation, which led to the inactivation of GSK3β. Moreover, GSK3β inactivation has been reported to be induced by limited nutrients (44). GSK3β inactivation via phosphorylation is commonly observed in cell senescence (45). The enhancement of glycogenesis triggered by inactivated GSK3β promotes cellular senescence and aging (46). The results from Fig. 1, *D* and *E*, suggest that glucose deprivation-mediated inactivation of GSK3β may be triggered by cell senescence. However, the inactivation of GSK3β by glucose deprivation did not lead to stabilization of β-catenin. A revealing observation herein is that the blocking of GSK3β with either siRNA or LiCl did not lead to attenuation of β-catenin degradation and autophagy in response to glucose deprivation, indicating that GSK3β is not required for glucose deprivation-induced autophagy in antagonizing the β-catenin pathway. Several reports indicated that a non-canonical Wnt signaling pathway can antagonize the canonical Wnt/β-catenin pathway (47). Specifically, Wnt5a was reported to promote GSK3β-independent degradation of β-catenin in the regulation of limb development (11). A non-canonical Wnt/Ca^2+^ pathway could also antagonize the canonical Wnt/β-catenin pathway (48). However, no expression changes in Wnt5a in response to glucose deprivation were detected herein (data not shown). These results strongly suggest that GSK3β and Wnt5a are not involved in the glucose deprivation-induced β-catenin degradation. Moreover, ERK activation triggered by growth factor/receptor or the hepatitis B virus-X protein (HBX) has also been reported to stabilize β-catenin and facilitate cell proliferation (49). However, the results obtained herein showed that U0126, a pharmacological inhibitor of ERK, could not attenuate the destabilization of β-catenin induced by glucose deprivation (Fig. 5*D*). Therefore, we turned our interest to PKC, because it can regulate autophagy activation or inhibition, according to previous studies. It has been shown that Ca^2+^-dependent PKC activation is required for autophagy in response to ER stress (50). In addition, palmitic acid induces autophagy in an mTOR-independent and PKC-dependent manner (51). Although Jiang *et al.* (52) showed conflicting findings that the inhibition of PKC increased autophagy, broad PKC activators or inhibitors were employed in that study. We considered that such indistinct target effects may not sufficiently reflect the PKCα-specific influence on autophagy during glucose deprivation. In that sense, PKC-dependent autophagic activation triggered by metabolic stress, such as glucose deprivation, provides reasonable evidence. Consistent with these studies, we found that the enhancement of LC3 protein levels by glucose deprivation was sufficiently blocked by PKCα inhibition. In addition, inhibition of PKCα, triggered by RNA knockdown or pharmacological inhibition, abolished glucose deprivation-induced β-catenin degradation. The precise mechanism by which PKCα is activated in response to glucose deprivation remains unknown. However, protein-protein interactions between PKCα and β-catenin exist under conditions of glucose deprivation, which suggests that glucose deprivation may enhance the interaction affinity between PKCα and β-catenin to promote the degradation of β-catenin. Taken together, our results demonstrated a molecular mechanism by which glucose deprivation could induce the GSK3β-independent degradation of β-catenin protein, leading to autophagy. The detailed molecular mechanisms in glucose deprivation, involving PKCα and β-catenin, needs further exploration in future studies.

## Footnotes

3 The abbreviations used are:

GSK3β: glycogen synthase kinase-3β
TCF: T cell-specific transcription factor
mTOR: mammalian target of rapamycin
LC3: microtubule-associated protein 1A/B-light chain 3
PI: propidium iodide
WCL: whole cell lysate.
